# Single-step 3D printing aided cranio-orbital reconstruction with patient specific polyetheretherketone implants after resection of benign spheno-orbital tumors

**DOI:** 10.1007/s00701-024-06393-4

**Published:** 2024-12-12

**Authors:** Arwin Rezai, Johannes P. Pöppe, Alexander Gaggl, Christoph J. Griessenauer, Christoph Schwartz, Herbert Krainz, Moritz Ueberschaer, Petra A. Mercea, Simon Enzinger

**Affiliations:** 1 Department of Neurosurgery, University Hospital Salzburg, Paracelsus Medical University, Salzburg, Austria; 2 Department of Oral and Maxillofacial Surgery, University Hospital Salzburg, Paracelsus Medical University, Salzburg, Austria

**Keywords:** Spheno-orbital meningioma, Cranioplasty, Orbital reconstruction, Polyetheretherketone, Patient specific implant

## Abstract

**Purpose:**

Computer-aided design (CAD) and computer-aided manufacturing (CAM) techniques have paved the way for single-step resections and cranio-orbital reconstructions with patient specific implants in spheno-orbital tumors. Here, we present our interdisciplinary maxillofacial and neurosurgical workflow and a case series of patients treated with this integrated approach.

**Methods:**

Patients, who underwent single-step resection of benign spheno-orbital tumors and cranio-orbital reconstruction with polyetheretherketone (PEEK) patient specific implants (PSI) from 2019 to 2024 in our institution were included. Three dimensional models of the tumor, the skull, the implants and the cutting guides were integrated into intraoperative neuronavigation and 3D printed at the point of care (POC) for surgical planning. Clinical data was retrospectively analyzed, pre- and postoperative Exophthalmic index (EI) was radiologically determined.

**Results:**

Eleven patients met inclusion criteria. Meningioma WHO grade 1 was the most common tumor entity (81.8%). In a majority of patients, exophthalmos was the presenting sign (63.6%). Postoperative cranial imaging revealed an optimal position of the PEEK implants with regredient EI in 88.9%. Four (36.4%) patients, of whom two (50%) had undergone prior tumor resections, suffered from surgical complications. The most commonly recorded complication was impaired wound healing (*n* = 2). Tumor recurrence was observed in one (9.1%) patient at six months follow-up.

**Conclusions:**

Single-step resection and reconstruction in spheno-orbital tumors with PEEK PSIs is feasible and combines surgical expertise, virtual implant design and 3D printing techniques. Favorable aesthetical, visual and oncological outcomes were achieved in this cohort, despite a significant risk for postoperative complications.

## Introduction

Neuro-oncologic pathologies with spheno-orbital infiltration are rare [12]. Adequate resection and reconstruction of the anterior skull base remains challenging because of the rather complex structure of the orbit and the risk for deterioration of visual acuity, due to intraoperative traction of the optic nerve. In a recent study, the importance of rigid orbital reconstruction and orbital volume for reduction Exophthalmus has been shown [14]. Rigid reconstruction of the orbit to avoid malalignment or a pulsating eye bulb is recommended, especially if the orbital roof and upper orbital rim have to be resected in addition to the lateral orbital wall [18]. Historically different materials have been used for skull reconstruction including bone grafts/substitutes, biomaterials and more recently computer-aided design (CAD) as well as computer-aided manufacturing (CAM) templates or implants [6, 16]. The distinct advantage of CAD/CAM implants is that they are tailored to the patient`s specific anatomy allowing for favorable aesthetics along with protection of the viscero- and neurocranium and prohibit possible comorbidity associated with autologous bone grafting [8, 18, 26]. Planning of those complex implants pre-operatively, however, remains a challenge as tumor margins and bone cuts have to be anticipated prior to surgery. Technologies like digital segmentation and planning software applications and 3D printing have recently enabled for a more advanced presurgical planning of resection margins and complex PSIs in the very sense of personalized medicine. Interdisciplinary cooperation of a specialized skull base team consisting of neurosurgeons and maxillofacial surgeons seems to be essential to optimize neuro-oncological tumor control, as well as functional, visual and aesthetical outcome.

A single-step reconstruction approach has been reported previously in case reports or case series [2, 3, 6–8, 11]. Here, we report a series of 3D printing aided single-step resections of spheno-orbital tumors and combined cranio-orbital reconstruction with CAD/CAM polyetheretherketone (PEEK) PSIs.

## Methods

**Fig. 1:**
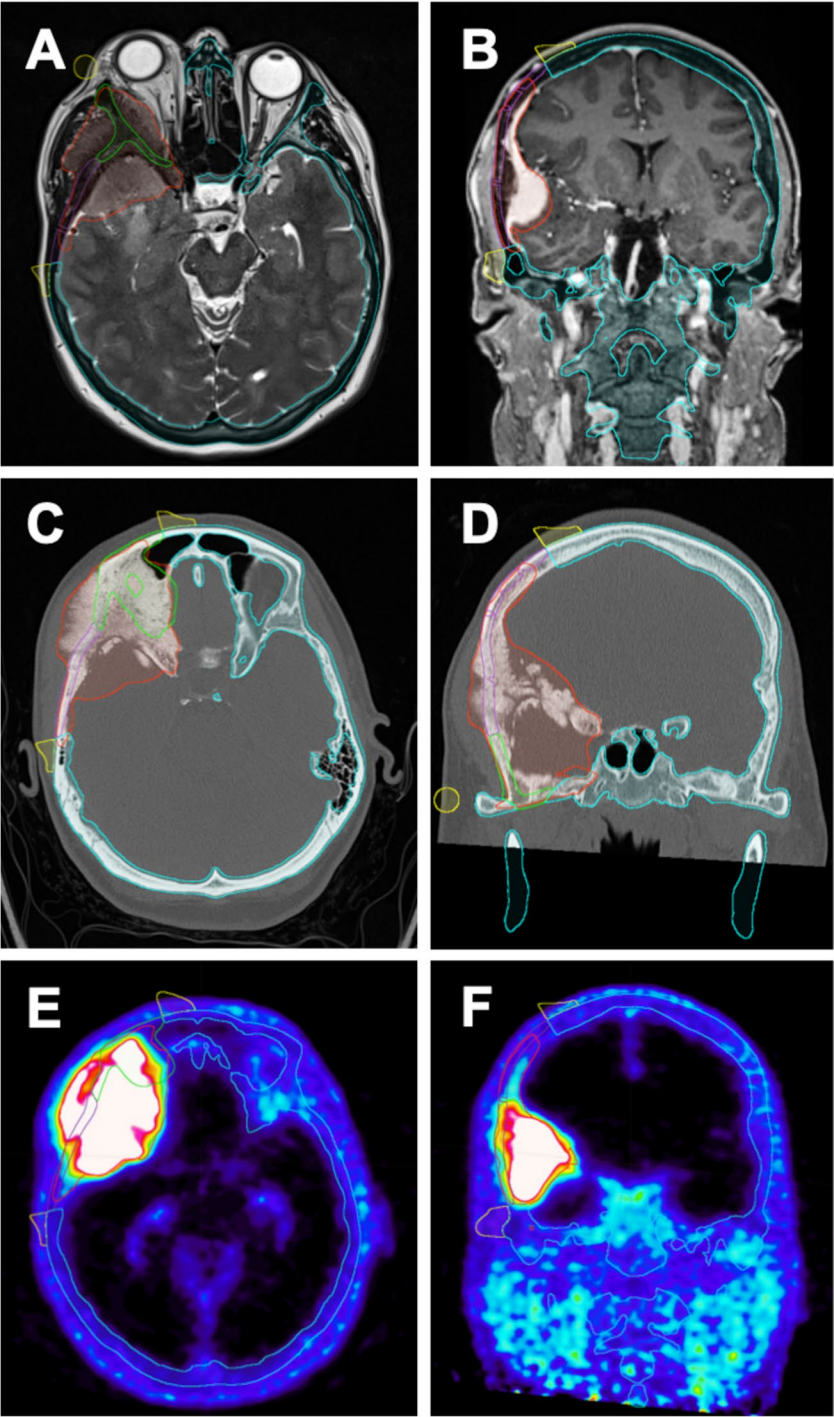
Preoperative virtual planning in Brainlab Elements software, using MRI T2 and contrast enhanced T1 sequences (**A**&**B**), cranial CT scans (**C**&**D**) and 68Ga-DOTATOC PET/CT scans (**E**&**F**). Tumor margins are defined by the treating surgeons (red lines), bony resection margins are defined (blue lines) and externally planned cutting guides (yellow lines) and implant margins (green lines) are later merged as STL files into the Brainlab planning (**A**-**F**)

**Fig. 2:**
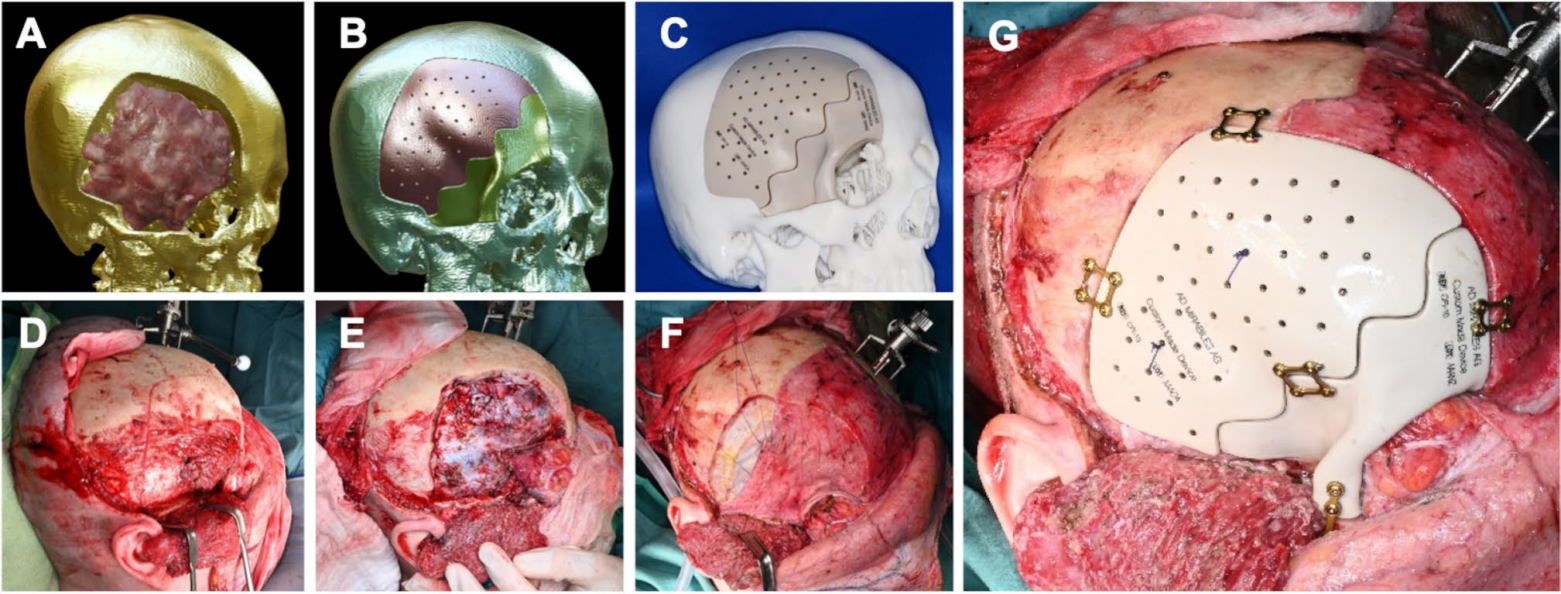
The workflow from virtual planning to implant insertion is shown in Fig. 2. 3-dimensional virtual planning shows tumor extention (red mass) and preplanned bony resection margins (**A**), the individually designed implants are previewed (**B**) and both, a template of the skull after craniectomy and the implants are 3D printed and used for preoperative and intraoperative planning (**C**). Intraoperative images show the marks for craniotomy on the skull (**D**), the skull after craniectomy with a thoroughly decompressed orbit (**E**) and dural closure with pericranium and Tutopatch (Biomedica, Milan, Italy) dural replacement (**F**). The final result shows optimal positioning of the two implants and fixation with titanium micro plates (**G**) (Medartis, Basel, Switzerland)

**Fig. 3:**
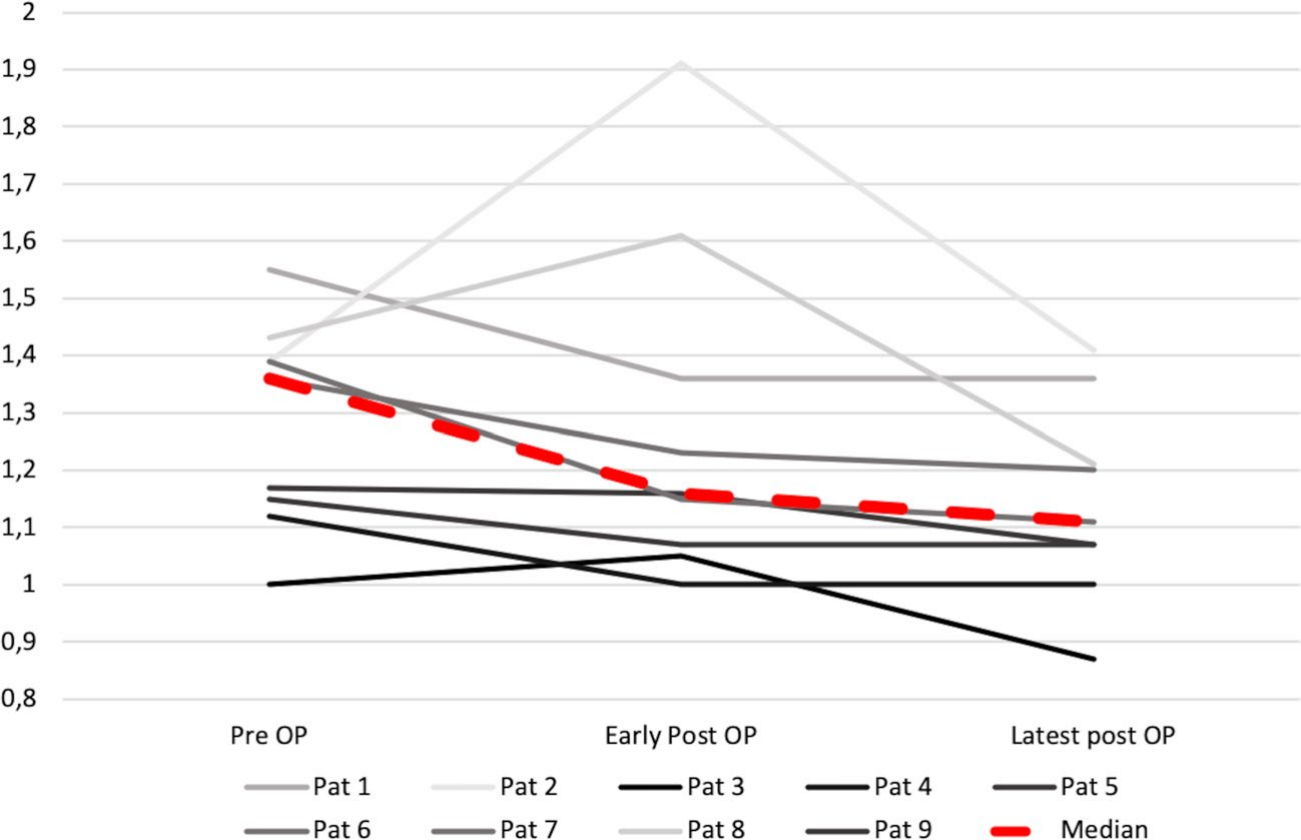
Development of Exophthalmic Index (EI) over time: a reduction of EI from median preoperative values of 1.36 (range 1–1.55) to latest postoperative values of 1.11 (range 0.87–1.41) is shown, with a reversible early postoperative increase of EI (median 1.16 [range 1–1.91]) in three patients

A cohort of consecutive patients diagnosed with benign spheno-orbital tumors, who received single stage cranio-orbital reconstruction with CAD/CAM PEEK PSIs between 2019 and 2024 were retrospectively analyzed. Demographic, functional and procedural data were extracted from the electronic medical records. All surgeries were conducted in a collaboration of the departments of oral and maxillofacial surgery and neurosurgery in a single academic teaching hospital. Presurgical planning was carried out on thin-sliced 1 mm computed tomography (CT) scans of the head and contrast-enhanced magnetic resonance imaging (MRI) of the brain. A subset of patients also received positron-emission-tomography CTs (68Ga-DOTATOC PET/CT). Based upon combined imaging modalities tumor and safe skull resection margins were defined in Brainlab Elements software (Brainlab AG, Munich, Germany) by the treating surgeons (Fig. 1). Individualized cutting guides and PEEK implants were designed by an external service provider (Ad Mirabiles LTD, Rheinfelden, Switzerland) in online real time consultation with the surgical team. Standard Triangle Language (STL) models of the tumor, the skull with resection margins, the implants and the cutting guides were integrated into intraoperative neuronavigation (Brainlab Elements) (Figs. 1 and 2A&B) and 3D printed with epoxy resins on a Formlabs Form 3B or 3BL stereolithography printer (Formlabs Inc., Somerville, MA, USA) at the point of care (POC) for surgical planning (Fig. 2C).

Prior to surgery written informed consent was obtained from all patients. Surgery was performed in accordance with institutional standards in a microsurgical fashion under general anesthesia. Implants were intraoperatively adapted in shape and size – for example by cutting the basal parts of the lateral orbital wall implant – if the preplanned resection margins could not be reached. Extent of resection and implant placement were verified with intra-operative flat panel imaging (Loop-X, MedPhoton, Salzburg, Austria/BrainLab, Munich, Germany). Postoperative imaging consisted of cranial MRI or CT scans on the first day after surgery and based upon histopathological findings after three to six months. Regular ophthalmological examinations of visual function and clinical follow up in the outpatient clinic were carried out. Based on preoperative, early postoperative and latest postoperative imaging (MRI or CT) Exophthalmic Index (EI) was calculated for all patients applicable [21].

All procedures described in this study were in accordance with the ethical standards of the state research committee (Ethics committee of the state of Salzburg) and with the 1964 Helsinki declaration and its later amendments or comparable ethical standards. A formal ethics committee approval was obtained (PMU-EK-2024-0007).

## Results

Between 2019 and 2024 resection of spheno-orbital tumors and single-step cranio-orbital reconstruction with PEEK PSIs was performed in eleven consecutive patients. Detailed characteristics of the patients are presented in Table 1. The median age was 52.2 years (range 19–72), all patients (100%) were females. Prior to the procedure, the most common presenting sign was exophthalmos in seven (63.6%) patients. Deterioration in visual acuity was seen in two (18.2%) patients. Another two (18.2%) patient had undergone orbital exenteration of the affected orbit before the index surgery. In total, four (36.4%) patients had undergone prior tumor resections, and another two (18.2%) patients had previous biopsies. Additionally, one (9.1%) patient had received radiotherapy at some point prior to the single-step resection.

**Table 1:** Characteristics and status of patients with spheno-orbital tumors treated by the single-step approach

	Patient 1	Patient 2	Patient 3	Patient 4	Patient 5	Patient 6	Patient 7	Patient 8	Patient 9	Patient 10	Patient 11
Sex	Female	Female	Female	Female	Female	Female	Female	Female	Female	Female	Female
Age at surgery	46	53	52	19	62	53	46	56	65	50	72
Previous surgeries	None	Biopsy	None	Biopsy	3	2	2	2	None	None	1
Histology	Meningioma*	Meningioma*	Meningioma*	Fibrous Dysplasia	Dermoid Cyst	Meningioma*	Meningioma*	Meningioma*	Meningioma*	Meningioma*	Meningeoma*
Leading signs or symptoms	Exophthalmos	Exophthalmos	Exophthalmos	Exophthalmos	Headache	Facial pain	Exophthalmos	Exophthalmos	Exophthalmos	Exophthalmos	Soft tissue swelling
EI prä OP	1,55	1,39	1	1,12	1,17	N/A	1,36	N/A	1,39	1,43	1,15
EI early post OP	1,36	1,91	1,05	1	1,16	N/A	1,23	N/A	1,15	1,61	1,07
EI last follow up	1,36	1,41	0,87	1	1,07	N/A	1,2	N/A	1,11	1,21	1,07
1-month postoperative leading symptoms	Improved	Improved	Improved	Improved	Improved	Unchanged	Improved	Improved	Improved	Improved	Worsened
Preoperative KPS	80	80	80	80	90	60	80	60	60	80	60
1-month KPS	90	90	90	60	100	60	70	70	70	90	50
6-months KPS	100	100	90	LTFU	100	60	80	80	90	Not reached yet	Not reached yet
Preoperative deterioration in vision	No	No	No	No	No	Yes	No	Yes	No	Yes	No
1-month postoperative vision	Unchanged	Unchanged	Unchanged	Worse	Unchanged	Unchanged	Worse	Unchanged	Unchanged	Improved	Unchanged
Postoperative complication	None	None	None	Intraorbital hematoma	Impaired wound-healing	None	Visual acuity deterioration	None	Impaired Wound healing	None	Pneumonia
Surgical site infection	No	No	No	No	Yes	No	No	No	No	No	No
Adjuvant therapy	None	None	Radiotherapy	None	None	Radiotherapy	None	None	None	None	None
Revision surgery	No	No	No	Yes	Yes	No	Yes	No	Yes	No	No
6-months tumor recurrence	No	No	No	LTFU	No	Yes	No	No	No	Not reached yet	Not reached yet

*KPS* Karnofsky performance status scale, *LTFU* lost to follow up, *N/A* not applicable (due to a planned orbital exenteration); *All Meningiomas were graded as central nervous system (CNS) World Health Organization (WHO) grade 1

Median incision-suture time was 781 min (mean 730,4 min.; range 231–1042 min.). In nine (81.8%) patients histopathological diagnosis revealed World Health Organization (WHO) grade 1 meningiomas, one recurrent epidermoid cyst and one fibrous dysplasia were the other diagnoses. Postoperative imaging showed an optimal position of the PEEK implants in all patients. Improvements of the leading clinical signs and symptoms were achieved in nine (81.8%) patients. Nonetheless, revision surgeries were necessary in four (36.4%) patients, due to an impaired wound healing in two (50%), a postoperative orbital hematoma in one (25%) and a postoperative visual acuity deterioration in one (25%) patient respectively. Adjuvant radiotherapy was applied in two (18.2%) patients because of relevant tumor remnants. Tumor recurrence six months after surgery was observed in one out of 8 (12.1%) patients who have reached the six months follow up.

Preoperative median Karnofsky performance status scale (KPS) was 80 (range 60–90). Six-months postoperative KPS of above 70 was reached in seven out of eight (87.5%) patients, a stable or improved KPS in all eight (100%) patients. One (9.1%) patient was lost to follow up and two (18.2%) patients did not reach the six months follow up yet.

Exophthalmic Index was determined in nine patients with a relevant exophthalmos (EI > 1.1) in eight (88.9%) patients. Median preoperative EI was 1.36 (range 1–1.55), early and latest postoperative median EI were 1.16 (range 1–1.91) and 1.11 (range 0.87–1.41) respectively (Fig. 3). EI was improved in eight (88.9%) patients in the latest imaging available with unchanged EI in one patient (11.1%). In two patients EI could not be determined due to prior orbital exenteration.

### Illustrative case (patient #1 in Table 1)

**Fig. 4:**
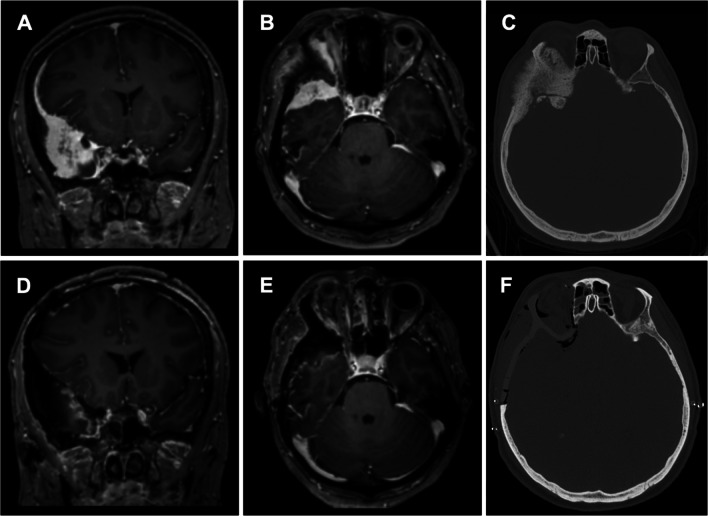
(patient #1) MRI of the head T1 sequences with contrast medium and thin-sliced CT scan of the head. A contrast medium enhancing lesion is seen at the right temporal pole (**A**) with infiltration of the orbital contents (**B**). In the bone window CT scan invasive growth into the frontal bone, the sphenoid wing, the zygomatic process and the lateral orbit is shown (**C**). Postoperative MRI scan with contrast medium reveals good tumor removal in the coronar (**D**) as well as in the axial view (**E**). CT scan of the head on the following day showed an optimal positioning of the PEEK implant with a good symmetrical result (**F**)

A 46-years-old female patient presented with exophthalmos, painful eye movement, and a sensation of retrobulbar pressure (Fig. 4). Ophthalmological examination was bilaterally intact. MRI of the head showed a homogenously contrast-enhancing tumor around the right temporal pole extending along the sphenoid wing down to the anterior clinoid with a dural tail sign in the frontal region and infiltration of the lateral orbital wall and the lateral rectus muscle (Fig. 4A&B). Preoperative Gallium 68 positron-emission-tomography CT showed pathologically increased signals in the right frontotemporal region, the right lateral orbital wall, the frontal bone, and the sphenoid wing with a maximum standardized uptake value of 25.3 (Fig. 1E&F). The cranial CT showed a sclerosing distention of the frontal bone with extension to the sphenoid wing, the zygomatic process, and the lateral orbit (Fig. 1C&D). Preoperative Planning, implant design and 3D printed templates are shown in Figs. 1 and 2A-C.

Surgical resection was performed through a bicoronal incision with pericranium harvest. The roof and walls of the right orbit were visualized. The zygomatic arch osteotomy was made at the level of the orbital floor. The temporal muscle was detached from the temporal bone and deflected caudally (Fig. 2D). The cutting guides were 3D printed preoperatively and screwed to the skull intraoperatively, guided by neuronavigation, to mark the resection margins with a piezo surgery instrument. A frontotemporal craniotomy was performed within the outlined craniotomy margins (Fig. 2E). Osseous tumor components were resected, the optic canal opened and the anterior clinoid process removed. Subsequently, dura and intradural tumor components were resected using microsurgical techniques. The frontal sinus was obliterated by using Tutopatch (Biomedica, Milan, Italy), TachoSil (Corza Medical, Linz, Austria), and pericranium (Fig. 2F). The PEEK PSI was fixated in two parts (orbit and cranial vault) with titanium cranial plates and 4 mm corticalis screws (Medartis Modus, Medartis AG, Basel Switzerland) (Fig. 2G). The position was controlled intraoperatively using Loop-X. Postoperative MRI imaging showed near-complete tumor resection with a potential small residual towards the cavernous sinus and optimal implant position (Fig. 4D-F). Ophthalmological examination at one and three months after surgery showed intact visual acuity and a significant decline of exophthalmos (EI improved from 1.55 to 1.36) with concomitant improvement of eye movement. The patient showed a full rehabilitation at six months follow up in the outpatient clinic. Esthetic outcome was improved over time with regredient ptosis and exophthalmos. Histological examination revealed a meningothelial meningioma WHO grade 1. A watchful waiting was recommended and the patient received further clinical and imaging follow up appointments.

## Discussion

Here, we provide our institutional experience of spheno-orbital tumor resections and single-step reconstruction using PEEK PSIs. More than half of all patients in our study showed a KPS improvement six months after surgery. One patient was lost to follow-up and two patients have not reached the six months postoperative mark.

According to the literature, the most common reason for lesions in the spheno-orbital region to require resection is exophthalmos with possible visual disturbances [1, 5, 9, 27]. The majority of lesions necessitating this approach in the present study were meningiomas. Although all meningiomas in this series were classified as WHO grade 1, their radiological and clinical growth patterns exhibit invasive growth and maximum safe resection is paramount [1].

### Advantages of the approach

While sufficient tumor resection and optical decompression is important, the aesthetic result matters for lesions that carry a long survival if managed correctly. The question if and how the lateral wall and the roof of the orbit should be reconstructed in spheno-orbital tumors remains a matter of debate. Nevertheless, a recent publication did show that rigid reconstruction of the orbit may lead to improved proptosis correction [14]. Whereas reconstruction of the orbit with bone grafts or standard-sized implants is still being used, the cosmetic and functional outcome of these patients may be suboptimal [8]. The implementation of CAD/CAM PSIs helps to improve the aesthetic and functional outcomes in patients with spheno-orbital lesion [22]. The single-step approach obliviates the need for additional surgery, which may result in the reduction of morbidity and the overall required hospitalization for patients, in those already complex and often multimodal treatment strategies [6]. PEEK facilitates imaging follow up as artifacts are minimized. However, the design of the patient-specific implant preoperatively requires meticulous planning. The implants have to be designed a priori anticipating the anatomical relationships even before any surgery. 3D printed models of the skull and the implants add a helpful dimension to the surgeon´s operative planning together with neuronavigation and the cutting guides. This leads to optimal PSI placement and thus thorough orbital and cranial reconstruction. Our findings are supported by a recent study that demonstrated improved proptosis correction in patients with spheno-orbital meningiomas if a rigid reconstruction was performed [14]. In our cohort an improvement of the leading symptoms was seen in nine (81.8%) patients with no change in one (9.1%) and worsened symptoms in another (9.1%) patient. The EI was improved or stable in all patients at last follow up, with a relevant reduction of median EI from 1.36 to 1.11, which is comparable to other series of spheno-orbital meningiomas [14, 21]. Thus, our approach is effective in reduction of exophthalmos, to preserve vision and for tumor control in benign lesions of the spheno-orbital skull base.

### Disadvantages and risks of the approach

Vision loss after orbital and periorbital surgery is a well-known complication with disastrous consequences. The incidence of this complication is the highest in orbital tumor resection compared to other orbital surgeries [13, 23]. In the current series, we had two patients (18.2%) who showed a deterioration in visual acuity postoperatively, which is in line with other studies, with most publications reporting visual deterioration in 15 to 25% of cases [1, 5, 18, 25]. The reasons for visual acuity deterioration in our series was an intraorbital hematoma in patient #4, which was evacuated secondarily. In patient #7 the most probable reason for worsened visual acuity was direct impairment of the optic nerve during optic decompression. To our knowledge, however, a single-step procedure with insertion of a CAD/CAM skull implant does not increase the risk of this complication [7].

Another concern is the risk of impaired wound healing and infection associated with alloplastic implants in general, which has led to the majority of complications in our series [10]. The high complexity of the combined reconstruction approach lead to an extensive median incision-suture time of 781 min. per patient, which harbors a likely risk factor for wound infections. Prior treatments complicate the management of these cases. Four (66.7%) out of six patients who did not receive prior treatment with intention of tumor resection showed immediate improvement of their symptoms without any postoperative complication. Higher complication rates were encountered in patients who had previous surgery or radiotherapy. For this reason, it may be advisable to pursue a single stage resection and reconstruction as the first intervention and not pursue a less invasive surgical treatment primarily, that will eventually require a complex reconstruction later on. One patient (patient #5) developed a wound infection with proven microbial growth in our study. She already received resection of a dermoid cyst in the fronto-orbital region 20 years prior to the single-step procedure in an external hospital. A small wound dehiscence with exposure of the implant was seen five months after the single-step procedure. The skin in this area was very thin, and likely a consequence of postoperative scarring from the surgical treatment 20 years before. In this case wound closure was performed utilizing a gracilis myocutaneous flap. Intraoperatively the implants were removed and reinserted after in-house sterilization in the same procedure. Four months after secondary surgery the patient was seen in the outpatient clinic with no remaining medical disturbances and a well-healed scar.

Complication rates are generally high in complex spheno-orbital tumor surgery, most commonly for meningeomas, independent from the used implant material. A large single center study on spheno-orbital meningeomas reported postoperative complications in 44% of patients with 26% of patients who had to undergo additional surgery for complication management [4]. In one of the largest published series on the resection of spheno-orbital meningeomas with 63 patients a total of 20 patients experienced surgical complications (32%), which resulted in nine secondary procedures, one patient remained dyspallic, and two patients died (3%). 10% of patients suffered from postoperative deterioration in visual acuity and 30% of patients had permanent postoperative cranial nerve deficits [18]. In these studies orbital reconstruction was – if performed at all – achieved with different implants and techniques. Thus, the fairly high overall complication rate with a revision rate of 36.4% in our study is comparable to other series in spheno-orbital tumors and should be considered in patient counseling and patient selection [5, 18].

### Future directions

PEEK implants have been shown to carry lower revision rates compared to other materials in cranioplastic surgery [6, 10, 15, 20, 24]. Future concepts will therefore focus on POC 3D printing of cranio-orbital PEEK PSIs, a technique that has been recently established by our 3D printing lab for large cranioplastic implants of the skull [17]. Thus, 3D printing together with virtual planning could become an integral part of cranio-orbital reconstructive surgery, with all necessary steps from implant design to implant insertion performed at the POC. Furthermore, the evolution of virtual reality and augmented reality based digital tools might replace physical cutting guides, which has been recently demonstrated for PEEK cranioplastic implants [19].

### Limitations

The study has several limitations. While the series consists of consecutive patients, data collection was performed retrospectively. Even though this is one of the largest series of spheno-orbital tumors treated with PEEK PSIs in the literature, the number of patients was small and allows for only descriptive analysis. Furthermore, all surgeries were performed by two maxillofacial surgeons (A.G., S.E.) in collaboration with several different neurological surgeons, which compromises the generalizability of quality of the surgical technique, since the impact of individual surgical quality on clinical outcome might be crucial.

## Conclusions

Interdisciplinary single-step tumor resection of skull base tumors with spheno-orbital involvement and implantation of a CAD/CAM PEEK PSI is feasible and combines surgical expertise, virtual implant design and 3D printing techniques. The approach allows for good aesthetical, visual and functional, as well as neurooncological outcome, despite a significant risk for postoperative complications, particularly in cases of prior treatment. In the future, we aim on developing a workflow to 3D print PEEK PSIs for cranio-orbital reconstruction directly at the POC as part of a more personalized medicine, which has already been proven to be feasible for large cranioplastic implants in our institution.

## Data Availability

No datasets were generated or analysed during the current study.
